# Distribution, structure, and mineralization of calcified cartilage remnants in hard antlers

**DOI:** 10.1016/j.bonr.2022.101571

**Published:** 2022-04-28

**Authors:** Uwe Kierdorf, Stuart R. Stock, Santiago Gomez, Olga Antipova, Horst Kierdorf

**Affiliations:** a Department of Biology, University of Hildesheim, Universitätsplatz 1, 31141 Hildesheim, Germany; b Department of Cell and Developmental Biology, Feinberg School of Medicine, and Simpson Querrey Institute, Northwestern University, 303 East Chicago Avenue, Chicago, IL 60611-3008, USA; c Department of Pathological Anatomy, University of Cádiz, Fragela 9, 11003 Cádiz, Spain; d X-ray Science Division, The Advanced Photon Source, Argonne National Laboratory, 9700 South Cass Avenue, Lemont, IL 60439, USA

**Keywords:** Antlers, Biological mineralization, Bone, Calcified cartilage, Endochondral ossification, Intralacunar mineralized deposits

## Abstract

Antlers are paired deciduous bony cranial appendages of deer that undergo a regular cycle of growth, death and casting, and constitute the most rapidly growing bones in mammals. Antler growth occurs in an appositional mode and involves a modified form of endochondral ossification. In endochondral bones, calcified cartilage is typically a transient tissue that is eventually completely replaced by bone tissue. We studied the distribution and characteristics of calcified cartilage in hard antlers from three deer species (*Capreolus capreolus*, *Cervus elaphus*, *Dama dama*), i.e., in antlers from which the skin (velvet) had been shed. Remnants of calcified cartilage were regularly present as part of the trabecular framework in the late formed, distal antler portions in all three species, whereas this tissue was largely or completely missing in the more proximal antler portions. The presence of calcified cartilage remnants in the distal antler portions is attributed to the limited antler lifespan of only a few months, which is also the reason for the virtual lack of bone remodeling in antlers. The calcified cartilage matrix was more highly mineralized than the antler bone matrix. Mineralized deposits were observed in some chondrocyte lacunae and occasionally also in osteocyte lacunae, a phenomenon that has not previously been reported in antlers. Using synchrotron radiation-induced X-ray fluorescence (SR-XRF) mapping, we further demonstrated increased zinc concentrations in cement lines, along the inner borders of incompletely formed primary osteons, along the walls of partly or completely mineral-occluded chondrocyte and osteocyte lacunae, and in intralacunar mineralized deposits. The present study demonstrates that antlers are a promising model for studying the mineralization of cartilage and bone matrices and the formation of mineralized deposits in chondrocyte and osteocyte lacunae.

## Highlights

• Remnants of calcified cartilage are regularly present in hard antlers of deer.
• Preservation of calcified cartilage is caused by the short lifespan of antlers.
• Calcified cartilage of antlers is more highly mineralized than antler bone.
• Mineralized deposits were observed in chondrocyte and osteocyte lacunae of antlers.
• SR-XRF showed increased Zn-concentration in cement lines and intralacunar deposits.

## Graphical abstract

**Figure:**
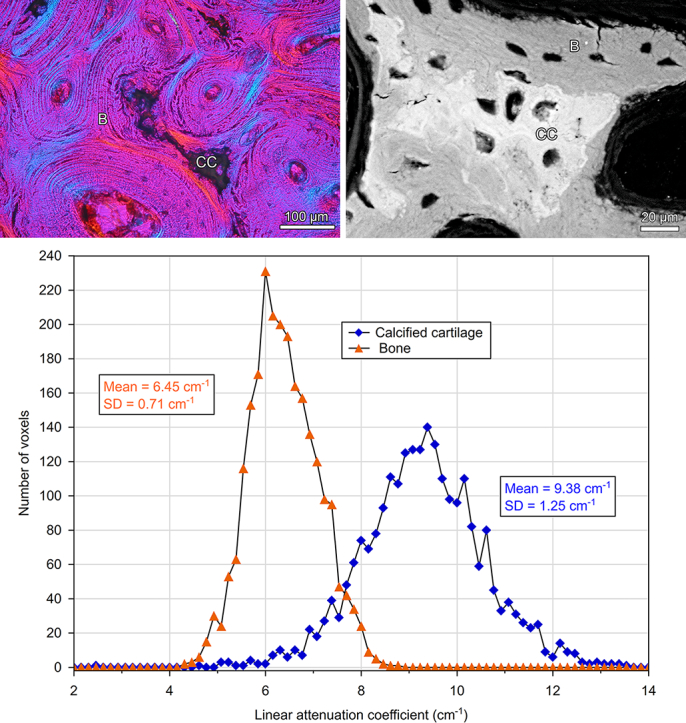

## 1 Introduction

The antlers of deer are annually replaced, paired bony appendages that develop on top of permanent outgrowths of the frontal bones, referred to as pedicles (Goss, 1983; Landete-Castillejos et al., 2019). Among extant cervids, only the water deer (*Hydropotes inermis*) lacks pedicles and antlers, and, except for the reindeer (*Rangifer tarandus*), antlers are normally grown only by males (Goss, 1983; Landete-Castillejos et al., 2019; Wang et al., 2019). While the pedicles are permanently skin-covered, the unique type of skin (velvet) that invests the growing antlers is shed after completion of growth, thereby exposing the bare bony (‘hard’ or ‘polished’) antlers. These are dead structures that are carried for a few more months, until they are detached (antler casting) and replaced by a new set of antlers (Goss, 1983; Currey et al., 2009; Gomez et al., 2013; Landete-Castillejos et al., 2019; Kierdorf et al., 2021). The timing of the antler cycle is under strict control of circulating androgens (Lincoln, 1992). Casting of the hard antlers is triggered by a marked drop in androgen levels, during antler regrowth androgen levels are low, and velvet shedding is caused by a steep rise in androgens.

Regenerating antlers constitute the fastest growing bones of mammals (Goss, 1983; Landete-Castillejos et al., 2019; Wang et al., 2019), and the periodic antler renewal within a few-month period is a unique example of extensive appendage regeneration in this taxon (Goss, 1983, Goss, 1984, Goss, 1987; Kierdorf et al., 2009; Kierdorf and Kierdorf, 2012; Li, 2012). The molecular characterization of the cartilage and other tissues from the antler growth regions, and the identification of the developmental pathways involved in the regulation of the stem-cell-based antler growth process are a matter of intense research (Kierdorf et al., 2009; Li, 2012; Zhang et al., 2018; Wang et al., 2019; Feleke et al., 2021; Chen et al., 2022). Of special interest is the finding that different proto-oncogenes are involved in antler formation, and that the expression profiles of growing antlers are more similar to those of osteosarcomas than to that of normal bone (Wang et al., 2019). Several tumor suppressor genes are, however, also highly expressed in growing antlers, leading to the conclusion that it is the unique combination of oncogenic and tumor-suppression pathways that enables rapid antler growth and simultaneously prevents tumorigenesis (Wang et al., 2019).

Antlers develop by a modified form of endochondral ossification, a process in which initially formed cartilage is replaced by bone (von Korff, 1914; Banks, 1974; Banks and Newbrey, 1983; Li and Suttie, 1994; Kierdorf et al., 1995; Price et al., 1996). The frontal bones of mammals are membrane bones derived from the neural crest (Gross and Hanken, 2008; Wu et al., 2017). The antler cartilage, which is a derivative of stem cells located in special regions of the frontal periosteum (Hartwig and Schrudde, 1974; Kierdorf and Kierdorf, 2012; Li, 2012), has been classified as a type of secondary cartilage (Galea et al., 2021). Antler elongation proceeds in an appositional mode by mesenchymal proliferation in apically located growth zones. Accordingly, growing antlers show a distal-to-proximal zonation along their vertical axis, with the youngest portions at the tips and the oldest at the base. The cartilage formed at the antler tips further proximally undergoes hypertrophy of its cells and mineralization (calcification) of its matrix (von Korff, 1914; Banks, 1974; Banks and Newbrey, 1983; Kierdorf et al., 1995; Price et al., 1996). In addition to longitudinal growth, there is also some circumferential growth of antlers by perichondral/periosteal bone apposition (Banks, 1974; Kierdorf et al., 1995; Price et al., 1996), a form of direct (intramembranous) ossification (Hall, 2015). This process causes the formation of a peripheral bone sleeve in the antler (Banks, 1974; Banks and Newbrey, 1983; Kierdorf et al., 1995, Kierdorf et al., 2007; Gomez et al., 2013; Kierdorf et al., 2013).

Antler cartilage is well vascularized and organized into interconnected vertical columns separated by perivascular connective tissue (Banks, 1974; Banks and Newbrey, 1983; Kierdorf et al., 1995; Landete-Castillejos et al., 2019). The rich vascularization of the antler cartilage is considered an adaptation to the rapidity of antler growth and its associated high metabolic demands (Goss, 1983). It has been hypothesized that antler chondrocytes, contrary to chondrocytes in the avascular growth plate, are not hypoxic (Galea et al., 2021).

As was already reported by von Korff (1914), the cartilage forms a largely tubular framework that runs parallel to the antler's long axis. About a century later, this view was reiterated by Krauss et al. (2011), who also characterized antler cartilage as exhibiting a longitudinally oriented tubular structure. Further proximally, the cartilaginous framework undergoes resorption by chondroclasts and is (at least partially, see below) replaced by an osseous one with the same orientation (von Korff, 1914; Banks, 1974; Banks and Newbrey, 1983; Li and Suttie, 1994; Kierdorf et al., 1995; Szuwart et al., 1998, Szuwart et al., 2002; Faucheux et al., 2001; Krauss et al., 2011; Landete-Castillejos et al., 2019).

In the future antler cortex, the intertrabecular cavities lined by the osseous framework (scaffold) are then filled with elongated primary osteons (Krauss et al., 2011; Gomez et al., 2013; Kierdorf et al., 2013; Skedros et al., 2014; Landete-Castillejos et al., 2019). The latter exhibit hypermineralized seams, or sheaths in three-dimensional perspective, along their periphery (Krauss et al., 2011; Kierdorf et al., 2013; Skedros et al., 2014). These seams represent cement lines that have been interpreted as either resting lines (Krauss et al., 2011) or reversal lines (Kierdorf et al., 2013). The latter view was based on observations indicating resorption at the bony scaffold prior to the infilling of the intertrabecular cavities with osteons (Gomez et al., 2013; Kierdorf et al., 2013). According to this interpretation, the latter partly replace previously existing bone (or calcified cartilage, see below) of the scaffold. This notwithstanding, these osteons have been classified as primary osteons as most of their volume occupies former soft tissue space (Kierdorf et al., 2013). In contrast, secondary osteons are formed by a coordinated process of osteoclastic resorption of (compact) primary or secondary bone and the subsequent infilling of the newly created spaces (resorption cavities) with lamellar systems (Burr and Akkus, 2014).

In endochondral ossification, bone is laid down on a scaffold of calcified cartilage (Allen and Burr, 2014). The mineral phase of this calcified cartilage (from the growth plates of 2-month-old calves) has been characterized as a poorly crystalline, immature apatite with a lower carbonate content than bone mineral (Rey et al., 1991). In mammals, the cores of calcified cartilage initially present in endochondral bone are typically completely replaced by bone tissue during remodeling, and calcified cartilage is therefore considered a (largely) transitory tissue in this taxon (Pritchard, 1972; Allen and Burr, 2014). There are, however, some exceptions to this rule. For example, a thin layer of articular calcified cartilage that is more mineralized than subchondral bone is regularly present in diarthrodial joints of the adult skeleton (Ferguson et al., 2003; Boyde, 2021). Persistence of calcified cartilage islands also occurs in long bones of rats and mice due to the lack of bone remodeling in these small mammals (Bach-Gansmo et al., 2013; Jing et al., 2017). An exceptional persistence of larger amounts of calcified cartilage among large mammals has been recorded in the ribs of extant and fossil sirenians (Fawcett, 1942; de Buffrénil et al., 2008) and fossil cetaceans (archaeocetes) (de Buffrénil et al., 1990). The condition is considered to result from a retardation/inhibition of chondroclastic activity and discussed as part of a specific skeletal adaptation to buoyancy control in these aquatic taxa (de Buffrénil et al., 1990, de Buffrénil et al., 2008).

Conflicting views exist with respect to the fate of the calcified cartilage in antlers. Banks (1974) and Banks and Newbrey (1983), who studied antlers of white-tailed deer (*Odocoileus virginianus*), mule deer (*Odocoileus hemionus*), red deer (*Cervus elaphus*), wapiti (*Cervus canadensis*), fallow deer (*Dama dama*) and reindeer (*Rangifer tarandus*), state that in the course of antler growth calcified cartilage and woven bone are eventually completely replaced by lamellar bone. Likewise, Krauss et al. (2011) suggested that during antler growth in red deer, the initially formed tubular framework of calcified cartilage is completely replaced by a scaffold of lamellar bone. They further reported that this lamellar bone shows a low degree of collagen orientation over longer distances and therefore referred to this tissue as micro-lamellar bone. Also Skedros et al. (2014) did not observe calcified cartilage in the studied regions of the hard antlers of mule deer, which however did not include the antler tips. In contrast, other authors have reported the persistence of remnants of calcified cartilage in hard antlers (especially their distal portions) of red deer (Landete-Castillejos et al., 2012), European roe deer (*Capreolus capreolus*) (Kierdorf et al., 2013), and fallow deer (Kierdorf et al., 2021).

The principal aim of the present study was to further analyze the distribution and structural properties of calcified cartilage in hard antlers of deer. We further compared the degree of mineralization of calcified cartilage and bone within the antlers and provide some data on calcium (Ca), phosphorus (P), and zinc (Zn) distribution within these hard tissues. The results from our study are consistent with the view that hard antlers are developmentally young structures that differ from mature somatic bone in several aspects.

## 2 Materials and methods

### 2.1 Antler samples

The studied antlers originated from three cervid species, the European roe deer (*n* = 5 individuals), fallow deer (*n* = 3), and red deer (*n* = 3). Except for the still growing, velvet-covered primary antlers of two yearling fallow bucks, all analyzed specimens were regenerated antlers whose velvet had been shed (i.e., hard antlers). The roe deer antlers had been collected from bucks killed during regular hunting operations for population management in Germany. This was the case also for the fallow buck whose hard antlers were studied. The growing primary fallow deer antlers and the hard antlers of the adult red deer stags were obtained from farmed animals slaughtered for venison using approved standard methods. The histological findings on the velvet-covered primary antlers of the fallow deer have been previously published (Kierdorf et al., 1995) and are re-used here for comparison. No animals were killed solely for collecting their antlers and therefore no special permits or ethical approval had to be obtained for this study.

### 2.2 Specimen preparation and optical microscopy

**Fig. 1:**
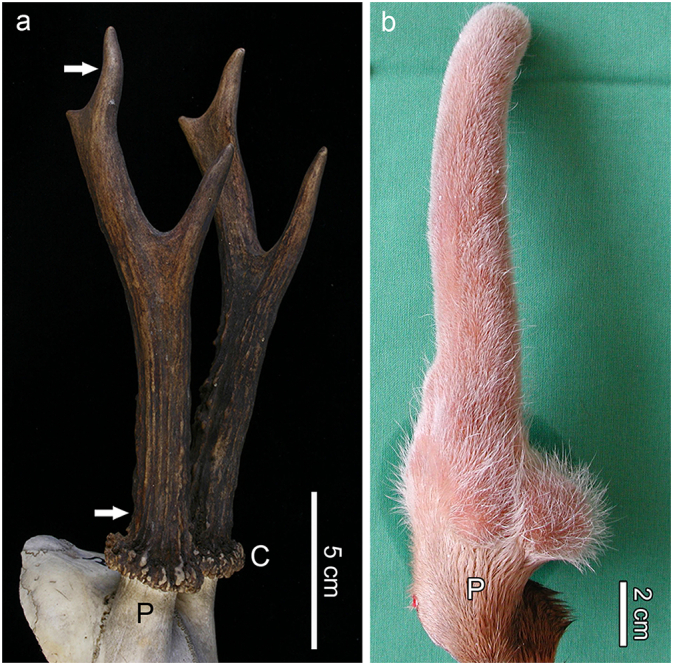
Overview images of a pair of regenerated hard antlers of an adult roe buck (a) and the external appearance of a primary velvet antler (plus pedicle) of a yearling fallow buck (b). Arrows in (a) indicate the approximate sampling locations of the transverse proximal and distal antler sections; P: pedicle, C: coronet (or burr). In (b), note the difference between the pedicle (P) covered by normal scalp skin and the antler covered by velvet.

Proximal and/or distal antler portions were sampled from the hard antlers of the three species (Fig. 1a). For that, either full-diameter transverse discs of 0.5 to 1 cm thickness were cut from the antlers with a fine-toothed saw, or the antler tips were removed and cut longitudinally. Several of the sampled antler portions were embedded in polymethylmethacrylate (PMMA, red deer antlers, one roe deer antler) or epoxy resin (Biodur®, antlers of roe deer and the hard fallow deer antler) as described previously (Landete-Castillejos et al., 2012; Kierdorf et al., 2013), while some hard antler samples from roe deer and fallow deer were studied un-embedded.

Transverse sections of about 50 μm or 15 μm thickness were prepared from PMMA-embedded antler samples of red and roe deer using a previously described grinding-polishing technique (Landete-Castillejos et al., 2012). Thick (50 μm) red deer antler sections were surface-stained with toluidine blue following acid etching (1% periodic acid) for 5 min. Thin (15 μm) sections of red deer antlers were decalcified and stained using a 2.5% solution of phosphomolybdic acid and phosphotungstic acid (Mo-W), and subsequently exposed to black (UV-A) light until they turned gray.

The surface-stained thick sections were studied by epifluorescence microscopy using an Optiphot-2 EFD 3 microscope (Nikon, Minato, Japan) with a cube filter V-2A. The decalcified and stained thin sections were examined in an Axioskop 2 Plus microscope (Zeiss, Oberkochen, Germany) and the Optiphot-2 microscope, using normal transmitted light (with phase-contrast enhancement, Axioskop) and linearly polarized light (LPL) with a 1λ retardation plate.

Fig. 1b shows the external appearance of a velvet-covered primary antler from a yearling fallow buck. Previously obtained microscopic images (Kierdorf et al., 1995) from longitudinal, 7 μm thick sections of paraplast-embedded, decalcified segments from the tip region of the growing primary antler of another fallow buck were used for comparison. The sections had been stained with the alcian blue – periodic acid Schiff (AB-PAS) method.

### 2.3 Preparation of block surfaces and SEM-BSE imaging

For backscattered electron (BSE) imaging of block surfaces of hard antlers, the cut surfaces of (embedded or unembedded) antler segments were smoothed and polished by hand using a series of silicon carbide papers (up to grit 4000), followed by a final polishing step on a motorized polisher (Labopol 5, Struers, Ballerup, Denmark) using first a diamond suspension of 3 μm particle size and subsequently an alumina slurry of 0.3 μm particle size. The uncoated samples were then studied (at 20 keV accelerating voltage) in low-vacuum mode in a scanning electron microscope (SEM, Evo Ma 15, Zeiss, Oberkochen, Germany), equipped with a high-definition thin five segment backscatter electron detector, with the polished surface oriented perpendicular to the primary electron beam. Gray level variation in the captured images reflects changes in local mineral content, with brighter gray levels characterizing areas of higher, darker gray levels areas of lower mineral content (Skedros et al., 1993; Roschger et al., 1998).

### 2.4 SEM-EDS analysis

Scanning electron microscopy-energy dispersive X-ray spectrometry (SEM-EDS) was performed on an epoxy resin-embedded polished block surface of a full-thickness transverse segment from the tip region of the hard fallow deer antler. Multipoint standardless analysis of Ca and P concentrations and mapping of the distribution of the two elements was done in the Zeiss Evo Ma 15 SEM (operated at 20 keV) using a Bruker XFlash 410-M detector (Bruker, Billerica, MA, USA). EDS data were collected and analyzed with the Quantax Esprit software (Bruker).

### 2.5 SR-μCT

Synchrotron radiation micro-computed tomography (SR-μCT) was performed on a pair of unembedded bars cut from the distal portion of a roe deer antler. The bars had widths of 1.15 mm along the antler's longitudinal axis and 0.95 mm along the transverse axis. The two similar bars from adjacent positions in the antler were packed in a plastic pipette tip and scanned simultaneously at beamline 2-BM of the Advanced Photon Source (APS), Argonne National Laboratory, Lemont, IL, USA. The particulars of the scans and reconstructions were: 22.7 keV photons, 1500 projections over 180°, 2400 × 2400 voxel reconstructed slices and 1.3 μm isotropic voxels. A stack of 151 slices was selected for analysis, and data on the volume fractions of bone, calcified cartilage and soft tissue spaces (voids, cell lacunae excluded) were obtained. In addition, linear attenuation coefficients (analyses of 2400 voxels each), as a measure of mineralization, were obtained for bone and calcified cartilage.

### 2.6 SR-XRF mapping

Synchrotron radiation-induced X-ray fluorescence (SR-XRF) mapping of Ca, P and Zn distribution and of transmitted intensity was performed on polished sections of unembedded and PMMA-embedded proximal and distal portions of hard roe deer antlers at beamline 2-ID-E, APS. The analyzed samples were scanned across the 13.4 keV beam whose dimensions were 400 nm vertical and 600 nm horizontal. The maps were recorded in the flyscan mode with 20 ms integration producing 300 nm × 300 nm pixels. The integrated K peak intensity was recorded.

## 3 Results

### 3.1 Calcified cartilage in growing antlers

**Fig. 2:**
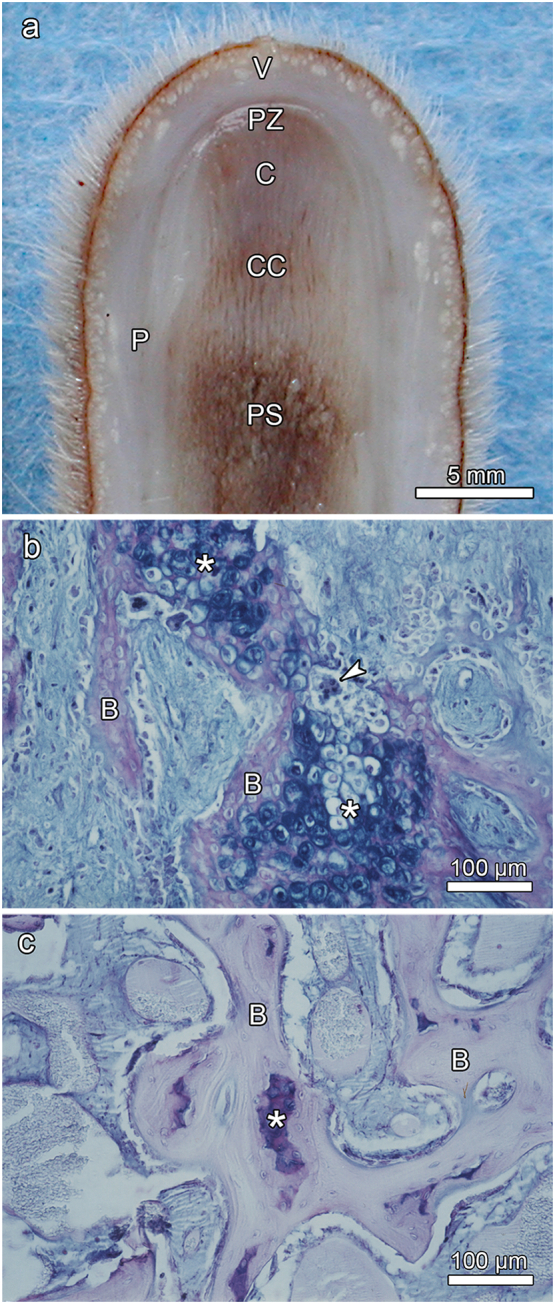
Longitudinal sections through the tip regions of growing primary antlers. (a) Macroscopic view of the sectioned tip of the fallow deer primary antler shown in Fig. 1; C: precartilage/mature cartilage, CC: calcified cartilage, P: periosteum/perichondrium, PS: primary spongiosa, PZ: proliferative zone, V: velvet. (b) and (c) Paraplast-embedded, AB-PAS stained sections (7 μm) through the tip region of another fallow deer spike antler viewed under normal transmitted light; reproduced with modifications from Kierdorf et al. (1995). (b) Resorption of calcified cartilage with hypertrophic chondrocytes (asterisks) by multinucleated chondroclasts (arrowhead) and concomitant deposition of woven bone (B) onto the surface of the cartilaginous trabeculae. (c) Further proximally, remnants of calcified cartilage (asterisk) are surrounded by bone (B), the more peripheral portions of which show a more lamellar organization. The calcified cartilage remnants exhibit scalloped borders from previous resorption.

In the growing fallow deer antlers (Fig. 1b), a zone of calcified cartilage was present below a zone composed of precartilage (distal portion) and mature cartilage (proximal portion). The precartilage was overlain by the proliferative zone (Fig. 2a). Further proximally, the calcified cartilage underwent resorption by chondroclasts, while simultaneously newly differentiated osteoblasts deposited woven bone onto the partially eroded, attenuated cartilaginous trabeculae (Fig. 2b). This zone of the growing antlers, the primary spongiosa (Fig. 2a), was thus characterized by the simultaneous occurrence of chondroclasia and osteogenesis. As a result of the combined chondroclastic and osteoblastic activities, the fraction of calcified cartilage became progressively diminished in proximal direction, while that of bone increased (Fig. 2c). The initially deposited bone that directly bordered on the calcified cartilage was woven bone (Fig. 2b), while later deposited, more peripheral bone showed a lamellar organization (Fig. 2c). Larger cores of calcified cartilage contained hypertrophic chondrocytes or their remnants, while in other places only thin streaks of mineralized cartilage matrix had been retained (Fig. 2c). The calcified cartilage remnants exhibited scalloped surfaces (Howship's lacunae) indicative of the previous resorption processes (Fig. 2c).

### 3.2 Calcified cartilage in hard antlers

**Fig. 3:**
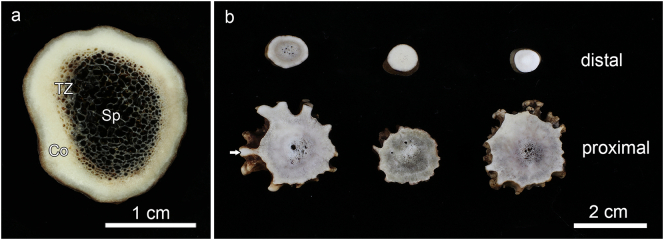
Macroscopic views of transverse sections of a hard antler from a fallow buck (a), sectioned about 7 cm above the coronet, and of transverse proximal and distal sections (section planes indicated in Fig. 1a) of three roe deer antlers (b). The fallow deer antler exhibits a compact cortex (Co), a cancellous spongiosa (Sp), and a narrow transition zone (TZ) between the two. The roe deer antlers show a higher proportion of compact bone than the fallow deer antler, and two of the three roe deer antler tips are entirely composed of compact bone. The arrow marks the cortical region depicted in Fig. 4c.

Morphologically, the hard antlers of the three studied deer species were basically composed of an outer zone of compact bone, the antler cortex, and an inner zone of cancellous bone, the antler spongiosa (Fig. 3 a). A relatively narrow transition zone of intermediate porosity was often present between cortex and spongiosa. The ratio of compact to cancellous bone was higher in the roe deer antlers compared to those of red and fallow deer (Fig. 3a, b). The very tip regions of the roe deer antlers were composed largely or entirely of compact bone (Fig. 3b).

**Fig. 4:**
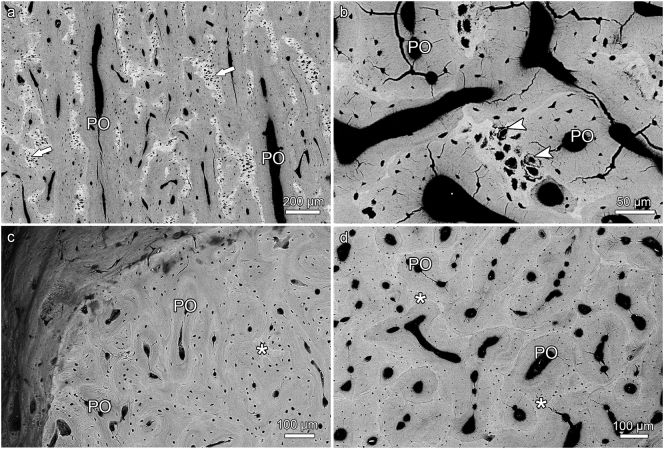
SEM-BSE images of polished block surfaces from unembedded portions of hard antlers from four roe bucks; for approximate location of samples see Fig. 1a. (a) Micrograph of compact bone from longitudinally sectioned antler tip, showing that larger amounts of calcified cartilage (arrows) have been retained within the scaffold. Note the more intense mineralization (brighter gray levels) of the mineralized cartilage matrix compared to the mineralized bone matrix, and the larger and rounder chondrocyte lacunae compared to the smaller and more lentiform osteocyte lacunae. (b) Micrograph of compact bone from a transversely sectioned antler tip, again showing the difference in matrix mineralization and lacunar size between calcified cartilage (higher brightness) and bone. Arrowheads: ring-like mineral deposits in chondrocyte lacunae, PO: primary osteons. (c) Micrograph of antler cortex from a transversely sectioned proximal antler portion in the region of a bone protuberance (“pearl”, location indicated by arrow in Fig. 3b). This antler portion is composed of bony scaffold (asterisk) and primary osteons (PO), while calcified cartilage is missing. (d) Micrograph of compact cortical bone from a transversely sectioned proximal antler portion. In this region, the antler is entirely composed of bony scaffold (asterisks) and primary osteons (PO). Note bright (hypermineralized) seams, interpreted as reversal lines, along the periphery of the osteons.

**Fig. 5:**
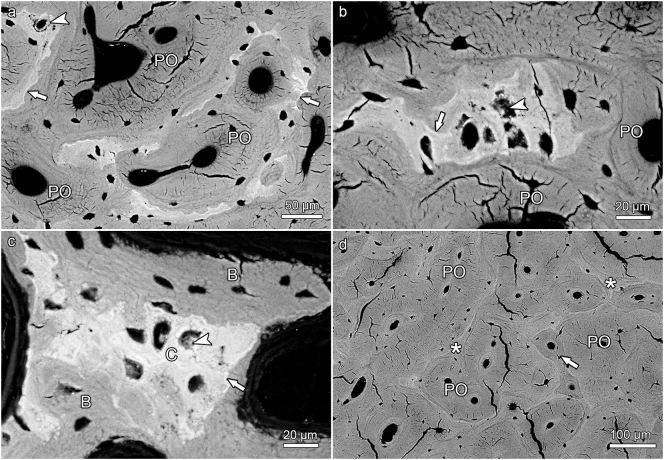
SEM-BSE images of polished surfaces from epoxy-resin embedded segments of the hard antler of a fallow buck. (a) Micrograph of portion of transversely sectioned distal antler cortex (3–4 cm below antler tip) showing remnants of calcified cartilage and primary osteons (PO). The difference in brightness denotes the more intense mineralization of the cartilage matrix compared to the bone matrix. Arrowhead: Ring-like mineral deposit in chondrocyte lacuna, arrows: hypermineralized seams, interpreted as reversal lines, along the margin of the calcified cartilage. (b) Detail of distal antler cortex showing a calcified cartilage remnant that exhibits a scalloped surface from previous resorption. Arrowhead: chondrocyte lacuna partly filled with mineral, arrow: hypermineralized seam (reversal line) along the margin of the calcified cartilage, PO: primary osteons. (c) Detail of distal antler spongiosa showing calcified cartilage (C) and bone (B). Arrowhead: partly mineral-occluded chondrocyte lacuna, arrow: hypermineralized seam (reversal line) along the margin of the calcified cartilage. (d) Micrograph showing portion of cortex from transversely sectioned proximal antler (main beam about 5 cm above brow tine). Asterisks: bony scaffold, PO: primary osteons, arrow: small secondary osteon (infringing on a PO). Note presence of hypermineralized seams (reversal lines) around osteons.

The antler cortex consisted of a predominantly tubular osseous framework and of primary osteons that had formed in the intertrabecular spaces of this scaffold (Fig. 4, Fig. 5, Fig. 7). Only few secondary osteons were encountered, and their presence was limited to the proximal antler portions (Fig. 5d). This finding indicates that the antler bone had not undergone any significant remodeling.

Remnants of calcified cartilage were non-uniformly distributed in the hard antlers of all three studied species. Thus, the proximal antler regions exhibited either no or (rarely) only very small remnants of calcified cartilage (Figs. 4c,d, 5d, 7d). Calcified cartilage was always absent from the peripheral cortex in the basal antler portions (Fig. 4c), which is consistent with the formation of these zones by direct ossification.

**Fig. 6:**
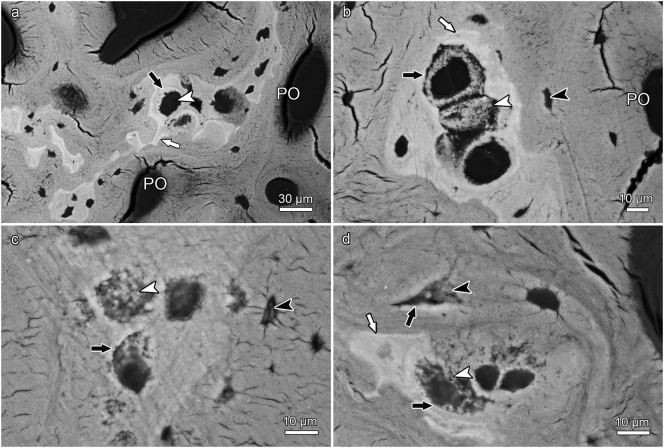
SEM-BSE images of polished surfaces from transverse sections of distal antler portions. (a) Transition zone between cortex and spongiosa of segment of the hard antler (PMMA-embedded sample) of a red deer stag. Remnants of calcified cartilage with scalloped surfaces. Note hypermineralized seams (reversal lines) along the margins of the calcified cartilage (white arrow) and hypermineralized rims (black arrow) along the borders of chondrocyte lacunae. Arrowhead: chondrocyte lacuna, PO: primary osteons. (b) Higher magnification of calcified cartilage remnant from the cortex of the hard red deer antler, showing chondrocyte lacunae filled with granular/globular mineral deposits (white arrowhead). The two upper lacunae are confluent, with peripheral ring-like mineralization in the upper one and an almost complete infilling in the adjacent one. Note hypermineralized seam (reversal line) along the surface of the calcified cartilage (white arrow) and hypermineralized rims along the borders of the chondrocyte lacunae (black arrow). Black arrowhead: osteocyte lacuna, PO: primary osteon. (c) Calcified cartilage remnant in the cortex of a hard antler from a roe buck (unembedded specimen), showing different degrees of infilling of the chondrocyte lacunae with granular/globular mineral deposits (white arrowhead). Black arrow: hypermineralized rim along border of chondrocyte lacuna, black arrowhead: mineral-occluded osteocyte lacuna. (d) Calcified cartilage remnant in hard antler cortex of a fallow buck. White arrowhead: chondrocyte lacuna partly occluded by mineral, black arrowhead: mineralized osteocyte lacuna, adjacent osteocyte lacuna not occluded, white arrow: hypermineralized seam (reversal line), black arrows: hypermineralized rims along borders of chondrocyte and osteocyte lacunae.

**Fig. 7:**
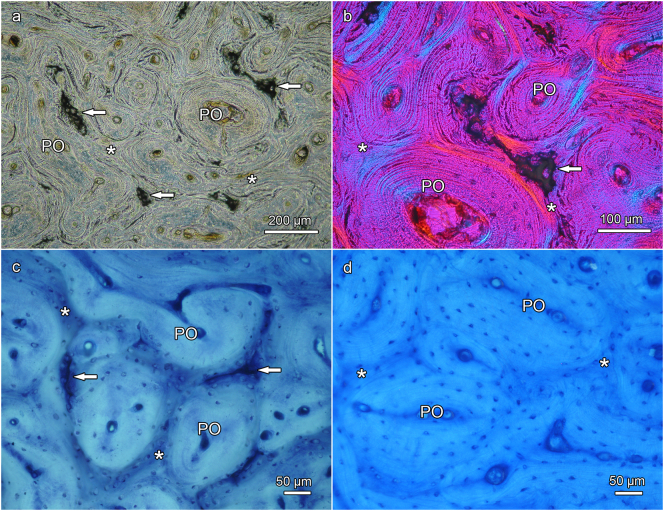
Light micrographs showing cortical bone from transverse ground sections through the distal third (a–c) and the proximal third (d) of hard antlers of red deer stags; PMMA-embedded specimens, (a) and (b) Mo-W stained thin sections (15 μm), (c) and (d) thick sections (50 μm) surface stained with toluidine blue. Arrows: remnants of calcified cartilage, asterisks: bony scaffold, PO: primary osteons. (a) The scaffold contains numerous remnants of calcified cartilage whose matrix appears dark; transmitted light with phase contrast. (b) Higher magnification showing larger area of calcified cartilage (with dark matrix) within the scaffold; linearly polarized light with 1λ retardation plate. (c) Remnants of calcified cartilage in the distal antler cortex exhibit a dark blue staining whereas bone stains light blue, normal transmitted light. (d) In the proximal antler cortex no remnants of calcified cartilage are present; normal transmitted light.

**Fig. 8:**
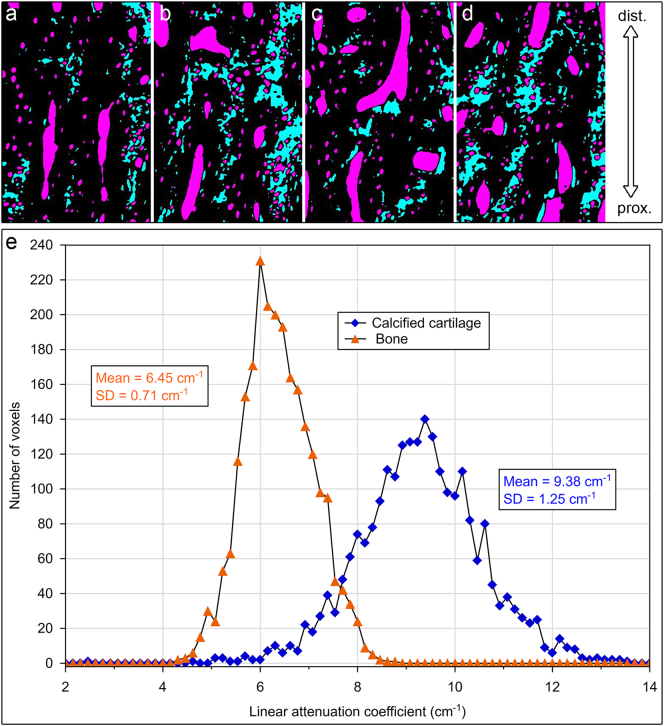
Results of SR-μCT analysis of a segment of an unembedded block cut from the tip region of the hard antler of a roe buck. (a–d) Color-coded images of four (of 151) μCT longitudinally oriented slices, showing the distribution of calcified cartilage (cyan), bone (black) and soft tissue spaces (magenta); 270 × 400 voxels per slice, field width of individual slice = 0.35 mm. Segmentation of the entire analyzed volume (stack of 151 slices) yielded volume fractions of 0.087 for calcified cartilage, 0.131 for soft tissue spaces (voids) and 0.782 for bone. (e) Histograms of linear attenuation coefficients of calcified cartilage and bone (analysis of 2,400 voxels each, 22.7 keV, 1.3 μm voxels) in the antler specimen.

In contrast to the proximal antler regions, remnants of calcified cartilage were regularly encountered as part of the trabecular framework in the distal antler regions of all three studied species (Figs. 4a,b, 5a-c, 6a-d, 7a-c). In some roe deer antlers, considerable portions of the trabecular framework from the tip regions consisted of calcified cartilage (Fig. 4a). More extended areas of calcified cartilage contained lacunae of hypertrophic chondrocytes that were distinguished from osteocyte lacunae by their larger size and more roundish shape (Figs. 4a,b, 5b,c, 6a-c). In places, confluent lacunae of adjacent chondrocytes were observed (Fig. 6b). Fig. 8a-d shows four color-coded images of slices (size of 270 × 400 voxels each) from the stack of 151 SR-μCT slices of a segment of the longitudinally sectioned distal roe deer antler. Segmentation of the entire analyzed volume yielded volume fractions of 0.087 for calcified cartilage, 0.131 for soft tissue spaces (voids) and 0.782 for bone. The original μCT-stack, the segmented slices, and a 3D-image of the entire stack are available for viewing in ImageJ (NIH, Bethesda, USA) as Supplementary Figs. 1, 2, and 3, respectively.

In the SEM-BSE images, the calcified cartilage matrix was characterized by brighter gray levels compared to bone, indicating its higher degree of mineralization (Figs. 4a,b, 5a-c, 6a-d). The higher mineral content of calcified cartilage compared to bone was also demonstrated by the difference in linear attenuation coefficients of the two tissues in the polished longitudinal section of the distal roe deer antler analyzed by SR-μCT (Fig. 8e). The mean value (± SD) for calcified cartilage was 9.38 ± 1.25 cm^−1^, that for bone 6.45 ± 0.71 cm^−1^ (air: 0.07 ± 0.31 cm^−1^). Variation in mineral concentration of the calcified cartilage (CV of 13.3% for the linear attenuation coefficient) exceeded that of bone (CV of 11.0%).

In areas where the calcified cartilage had been more extensively resorbed, often only thin streaks of mineralized cartilage matrix had remained (Fig. 5a). The surface of the calcified cartilage remnants was scalloped due to the presence of Howship's lacunae (Figs. 4b, 5a-c, 6a,b, 7a,b), indicating intense previous resorption activity. In the SEM-BSE images, hypermineralized (bright) seams were observed along the scalloped borders of the calcified cartilage (Figs. 5a-c, 6a,b,d). These seams are interpreted as cement lines deposited onto reversal surfaces (i.e.*,* reversal lines) of the calcified cartilage. In places, primary osteons directly bordered on the calcified cartilage (Figs. 4b, 5a,b). Hypermineralized cement (reversal) lines were also observed along the periphery of primary osteons in the proximal antler cortex (Figs. 4d, 5d), denoting resorption at the trabecular scaffold prior to infilling of the intertrabecular spaces with primary osteons.

**Fig. 9:**
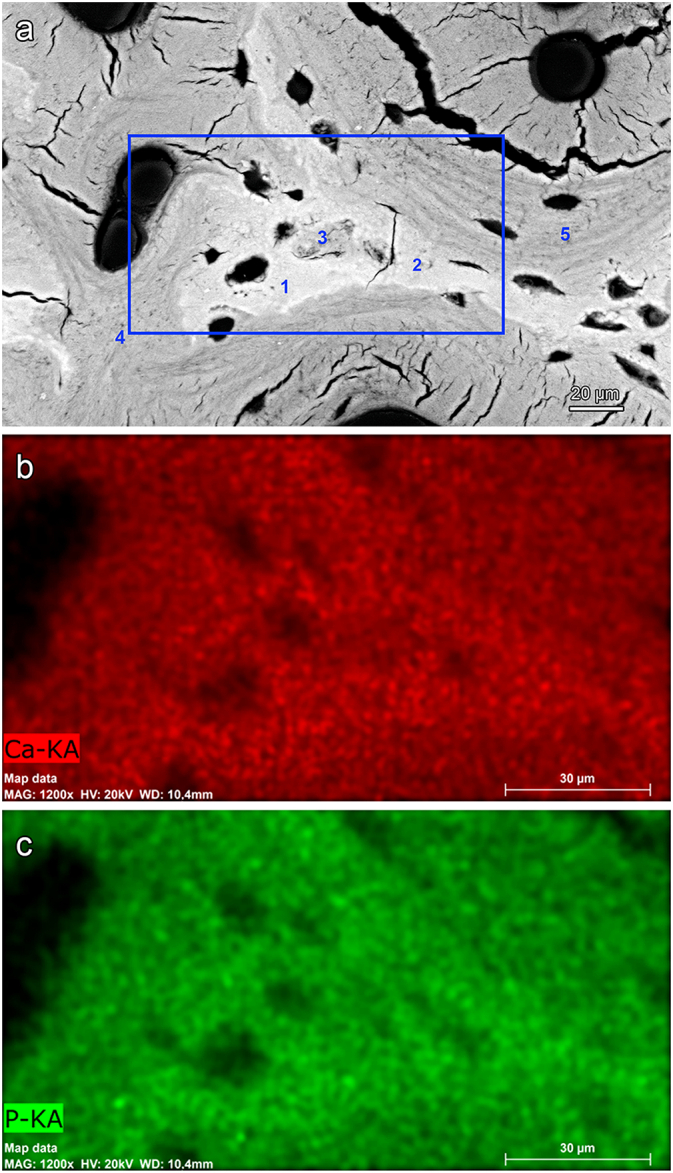
SEM-BSE image of the antler cortex (polished block surface of epoxy-resin embedded specimen) from the transversely sectioned distal portion of a hard fallow deer antler (a), and SEM-EDS maps showing the distribution of calcium (b) and phosphorus (c) in the area indicated by the rectangle in (a). The numbers in (a) indicate the location of the measuring spots of the multipoint analysis whose results are given in Table 1. The higher Ca and P intensities (brighter color = higher intensity) indicate higher concentrations of both elements in calcified cartilage than bone, which is also demonstrated by the multipoint analysis. Note an apparently completely mineral-occluded chondrocyte lacunae marked by “3” in (a).

The SEM-BSE images further demonstrated the presence of mineralized deposits in some of the chondrocyte lacunae of the calcified cartilage remnants (Figs. 4b, 5 a-c, 6a-d, 9a). The deposits partly (Figs. 4b, 5a-c, 6d) or completely (Figs. 6c, 9a) filled the lacunae and often exhibited a granular to globular texture (Fig. 6b-d). In the case of incompletely filled lacunae, the mineralized deposits frequently showed a ring-like shape (Figs. 4b, 5a, 6b). The brightness of the intralacunar deposits indicated a mineral content similar to that of the surrounding mineralized cartilage matrix (Figs. 4b, 5c, 6b-d, 9a). In the SR-μCT image stack, mineralized deposits could also be observed in chondrocyte lacunae located deeper within the analyzed sample that had no connection to the specimen surface. This excludes that the intralacunar deposits were artefacts from the polishing process. Mineralized deposits were occasionally also observed in osteocyte lacunae adjacent to calcified cartilage remnants (Fig. 6c,d). The partly or completely occluded chondrocyte lacunae frequently exhibited bright (hypermineralized) rims along their borders (Fig. 6a-d). Occasionally, such hypermineralized rims were also observed along the borders of osteocyte lacunae filled with mineral deposits (Fig. 6d).

In the Mo-W stained thin antler sections from the red deer, the mineralized cartilage matrix appeared dark (higher refractive index compared to the surroundings) when examined by phase-contrast microscopy (Fig. 7a). It also appeared dark (black interference color) when studied with LPL using a 1λ plate (Fig. 7b). In the thick sections that had been surface-stained with toluidine blue, the mineralized cartilage matrix was identified by its intense staining (Fig. 7c).

### 3.3 Elemental analysis and mapping

**Fig. 10:**
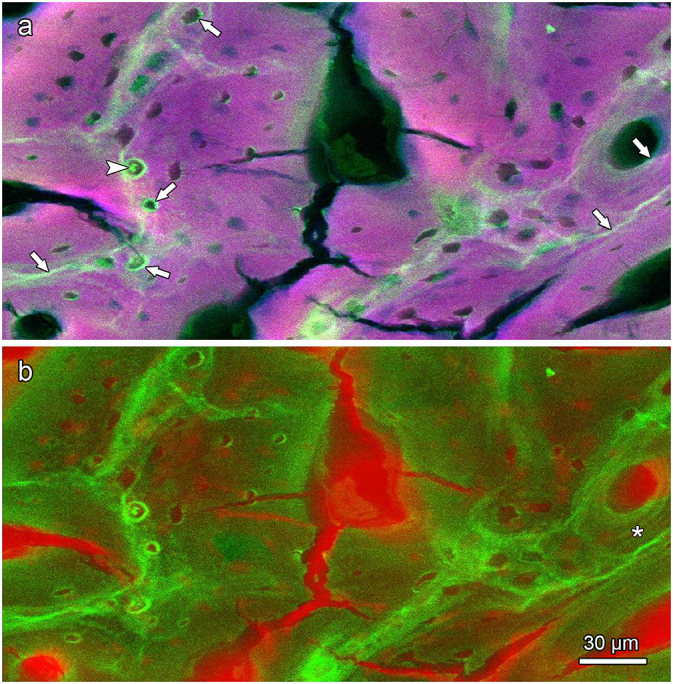
SR-XRF maps showing distribution of calcium, phosphorus, and zinc in the transversely sectioned tip (thin section, 15 μm) of the hard antler of a roe buck. Magenta color in (a) indicates the combined Ca and P intensities (brighter color = higher intensity), red color in (b) indicates transmitted intensity, and green color in (a) and (b) indicates Zn intensity. There is a higher zinc intensity in the (hypermineralized) reversal lines around primary osteons (arrows), indicating Zn-enrichment in these lines compared to the surrounding mineralized matrix. Increased Zn-levels were also observed at the margins of chondrocyte lacunae and along the borders of the vascular canals of the primary osteons (arrows). Occasionally, the latter show concentric bands of strong versus low Zn intensity that reflect the lamellar architecture of the osteons and are interpreted as signal of the rhythmic growth/mineralization process of these structures. The intralacunar mineral deposits observed in some of the chondrocyte lacunae (arrowhead) also show a high Zn-content.

SEM-EDS multipoint analysis and elemental mapping of the polished sample from the antler tip region of the hard fallow deer demonstrated higher Ca and P concentrations in mineralized cartilage matrix and mineralized chondrocyte lacunae compared to the surrounding mineralized bone matrix (Fig. 9b,c, Table 1). A higher Ca and P concentration of antler calcified cartilage compared to bone was also indicated by SR-XRF mapping of a transverse section from the distal portion of a hard roe deer antler (Fig. 10a). This mapping further demonstrated an increased Zn content in the hypermineralized cement (reversal) lines compared to the surrounding mineralized matrix (Fig. 10b). Higher Zn intensities were also observed along the borders of the vascular canals of primary osteons with rather wide osteonal canals (Fig. 10b). A particularly high Zn intensity was furthermore recorded along the borders of the chondrocyte lacunae, corresponding to the hypermineralized rims observed by SEM-BSE imaging, and for the mineralized deposits that had filled these lacunae (Fig. 10b). Occasionally, the primary osteons exhibited concentric bands of stronger versus lower Zn intensity that resembled the lamellar architecture of the osteons (asterisk in Fig. 10b).

**Table 1:** Results of the SEM-EDS multipoint measurements of calcium (Ca) and phosphorus (P) concentrations (wt%, normalized values) and Ca/P ratios in calcified cartilage and bone from the tip of a hard fallow deer antler. For location of the measuring spots see Fig. 9a.

Measuring spot	Location	Ca (wt%)	P (wt%)	Ca/P ratio
1	Mineralized cartilage matrix	29.44	12.63	2.33
2	Mineralized cartilage matrix	27.68	12.09	2.29
3	Mineralized chondrocyte lacuna	27.79	11.94	2.33
4	Mineralized bone matrix	23.55	10.08	2.34
5	Mineralized bone matrix	23.02	9.87	2.33

## 4 Discussion

The present study demonstrated the regular presence of calcified cartilage remnants in the distal portions of hard antlers from all three studied species, thereby confirming the results of previous studies (Landete-Castillejos et al., 2012; Kierdorf et al., 2013, Kierdorf et al., 2021). In the late-formed, distal antler regions, resorption of the framework of calcified cartilage was thus typically incomplete, while further proximally the cartilage was largely or entirely replaced by bone in the course of antler histogenesis. The observations that, in the antler tip regions, primary osteons sometimes directly bordered on the calcified cartilage suggests that resorption of the calcified cartilage scaffold had in places been directly followed by primary osteon formation, without an intervening phase of replacement of the cartilaginous by an osseous trabecular framework. This is seen as indicative of a shortened duration of the successive histogenetic phases in the distal compared to the more proximal antler portions, with the occasional skipping of an entire step (replacement of the cartilaginous by an osseous scaffold) in the ossification process.

In the present study, the calcified cartilage remnants within the osseous framework of hard antlers could be readily diagnosed by different methods (light microscopy, SEM-BSE imaging, SR-μCT). The fact that other authors (Banks, 1974; Krauss et al., 2011; Skedros et al., 2014) do not report the presence of such remnants may be due to the antler sampling schemes in these investigations. Based on the information given in the papers, it appears that none of the above studies analyzed samples from the tip regions of hard antlers.

Our finding of a more variable mineral content of antler calcified cartilage compared to bone matches previous observations on human calcified cartilage and bone (Ferguson et al., 2003; Lau et al., 2011). We further show that the residual cartilage matrix of hard antlers is more intensely mineralized than the surrounding bone matrix. A higher mineral content of articular calcified cartilage compared to subchondral bone was previously observed in femoral heads of humans (Ferguson et al., 2003). A more intense mineralization of calcified cartilage compared to bone has also been reported for mandibles of human infants (Goret-Nicaise and Dhem, 1985) and hind-limb elements of rats and mice (Bach-Gansmo et al., 2013; Jing et al., 2017). In contrast, Lau et al. (2011) found no significant difference in average mineral density between calcified costal cartilage and rib bone of adult humans; however, the maximum value of the calcified cartilage exceeded that of bone.

The presence of hypermineralized seams (cement lines) along the margins of remnants of calcified cartilage has previously been observed also in mature midfemoral rat bone (Bach-Gansmo et al., 2013). It has been discussed whether these hypermineralized cement lines may function in fracture deflection, which would increase the toughness of certain bone areas (Bach-Gansmo et al., 2013). These authors, however, also argue that the calcified cartilage islands can over time accumulate microdamage, thereby actually reducing rather than increasing bone toughness. Based on the latter reasoning, it has been hypothesized that an additional role of bone remodeling in large animals could be the removal of such calcified cartilage islands in endochondral bone (Bach-Gansmo et al., 2013).

The persistence of calcified cartilage in the distal antler regions and the lack of any significant remodeling of the antler bone are regarded as the inevitable consequence of the short (only few months) lifespan of antlers (Kierdorf et al., 2021). The latter view is supported by the observation (in fallow deer) that when the lifespan of antlers is experimentally extended by antiandrogen treatment, the calcified cartilage in the antler tips is completely replaced by bone in the course of marked remodeling (Kierdorf et al., 2021). Further evidence of this process was the frequent presence of secondary osteons and resorption cavities in the antler bone of the experimental animals. Interestingly, the presence of remnants of hypertrophic cartilage was recently reported in the antler tip regions of Miocene deer (*Procervulus praeludicus* and *Lagomeryx parvulus*) (Rössner et al., 2021). This was interpreted as evidence of a short antler lifespan also in these early cervid species, corresponding to the situation in extant deer.

To the best of our knowledge, mineral deposits in the chondrocyte lacunae of antler calcified cartilage are here described for the first time. Intralacunar mineral deposits have previously been observed in human laryngeal cartilage (Pascher, 1923) and recently also in the calcified cartilage of extinct and extant birds (Bailleul and Zhou, 2021). The sometimes ring-like appearance of the deposits in the antlers' chondrocyte lacunae suggests a centripetal infilling process, which could reflect the shrinking of a dying chondrocyte and the related expansion of the pericellular space within a lacuna. In human articular cartilage, chondrocyte apoptosis is associated with degradation of the pericellular matrix and the formation of apoptotic bodies (Hashimoto et al., 1998). We observed mineral deposition also in a few osteocyte lacunae of the studied antlers. Mineralization of osteocyte lacunae has previously been reported from other bones and is referred to as micropetrosis (Frost, 1960; Boyde, 2003; Bell et al., 2008; Milovanovic and Busse, 2020). It is generally agreed that it is associated with osteocyte apoptosis (Milovanovic and Busse, 2020), and lacunar mineralization has been characterized as the most solid evidence of osteocyte death (Boyde, 2003).

In humans, partially or completely mineral-occluded osteocyte lacunae are most often found in aged and osteoporotic bone (Milovanovic and Busse, 2020). Some observations suggest that the occlusion of osteocyte lacunae with mineral may be preceded by mineralization of canaliculi and that sometimes the dying osteocyte or the “cell mummy” itself can become mineralized (Boyde, 2003; Bell et al., 2008). A similar phenomenon was previously also reported for human chondrocytes (Pascher, 1923). Mineralization of osteocyte lacunae often occurs via the formation of spherical bodies termed nanospherites that have been characterized as representing mineralized apoptotic cell debris (Boyde, 2003; Bell et al., 2008; Milovanovic et al., 2017; Milovanovic and Busse, 2020). The granular/globular mineral deposits in chondrocyte and osteocyte lacunae of antlers resemble such deposits. The mineralization of chondrocyte lacunae in antlers is therefore supposed to be associated with the apoptosis of hypertrophic chondrocytes that has been observed to occur during antler growth (Szuwart et al., 1998).

Living osteocytes are able to maintain an unmineralized pericellular space by producing crystallization and mineralization inhibitors and enzymes (such as matrix metalloproteinases) that digest their immediate surroundings (Milovanovic and Busse, 2020 53). When the osteocyte dies, this inhibition is no longer effective and the lacuna can undergo passive (spontaneous) mineralization (Milovanovic and Busse, 2020). It has, however, further been shown that dying osteocytes can release matrix vesicles, suggesting the possible involvement also of an active component in lacunar mineralization (Milovanovic et al., 2017; Milovanovic and Busse, 2020).

The bone mineral in the micropetrotic osteocyte lacunae is a hydroxyapatite, and the mineralized lacunae show a higher mineral to matrix ratio than the surrounding bone matrix (Milovanovic et al., 2017). Similarly, our SEM-EDS findings indicated a higher Ca and P content of the mineral deposits within the chondrocyte lacunae of hard antlers compared to the mineralized bone matrix. It is currently unknown if the mineralization processes in chondrocyte and osteocyte lacunae of antlers takes place simultaneously or at different times.

Our finding of Zn enrichment in cement lines of the hard antlers matches previous results obtained on human and bovine bone using both SR-μXRF mapping and histochemical methods (Gomez et al., 2006; Pemmer et al., 2013). The increased Zn level in cement lines has been attributed to Zn-ions incorporated in the hydroxyapatite and/or to Zn containing proteins that are buried in the cement lines during the remodeling process (Everts et al., 2002; Pemmer et al., 2013). Zn-distribution in the antlers could be visualized by SR-XRF, but not by SEM-EDS due to the lower sensitivity of the latter method. Thus, previously reported Zn-concentration in antler bone (Kierdorf et al., 2014; Giżejewska et al., 2016; Gomez et al., 2016; Landete-Castillejos et al., 2019) are far below the detection limit in SEM-EDS measurements of about 0.1 wt% (Reed, 2005).

In samples of porcine osteonal bone subjected to different Zn-extraction procedures, Gomez et al. (1999) identified three zinc pools, viz., a loose, a mineral-bound, and a tenacious one. The latter zinc was tightly bound to the organic matrix. Loose zinc was found at the inner layer of the osteoid seam of forming osteons, while mineral-bound zinc was present at the mineralization front and at cement lines. The co-localization of tenacious zinc with alkaline phosphatase (ALP) protein suggests that this zinc corresponds to the functional zinc of this metalloenzyme. Tenacious zinc and ALP protein were detected only in growing but not in completed osteons (Gomez et al., 1999).

In line with these earlier findings, a later study on antler bone of red deer found higher Zn levels at the mineralization front of forming primary osteons with still wide osteonal canals and thick osteoid seams (Landete-Castillejos et al., 2012). In the present study, Zn enrichment was likewise observed along the osteonal borders of primary osteons with wide osteonal canals from roe deer antlers by SR-XRF. In keeping with the interpretation given by Landete-Castillejos et al. (2012), we conclude that these osteons were still forming when the antler died off at or shortly after velvet shedding. We presume that the high Zn content along the osteonal border reflects the presence of metalloenzymes (such as ALP) in these locations. The origin of the high Zn levels in the mineralized intralacunar deposits and along the (hypermineralized) walls of the chondrocyte lacunae in the antlers remains to be determined. They could at least partly represent ALP and/or trapped zinc-containing matrix metalloproteinases (Verma and Hansch, 2007).

Elevated Zn concentrations are often associated with ongoing biomineralization (Naji et al., 2022) and have been demonstrated to be preserved in structures such as cementum and dentin growth bands (Stock et al., 2017; Ryan et al., 2020). A rather faint (background) Zn staining of lamellar structures of primary osteons that resembles our findings in the roe deer antlers (Fig. 9) was previously also observed in red deer antlers (Landete-Castillejos et al., 2012). Further studies comparing the distribution of zinc in antler and pedicle bone of deer are encouraged.

In conclusion, the present study has demonstrated that remnants of calcified cartilage are regularly present in the distal antler portions of all three studied deer species. The persistence of calcified cartilage in antlers reflects their short lifespan of only a few months that does not allow for complete resorption of calcified cartilage and its replacement by bone in the youngest antler regions. The short-lived nature of antlers is also the reason for the absence of any significant remodeling of antler bone (Kierdorf et al., 2021). Our analysis confirmed that the calcified cartilage in antlers is more highly mineralized than the bone matrix. We further demonstrated, for the first time, the occurrence of mineralized deposits within chondrocyte lacunae of the calcified cartilage in roe deer, red deer, and fallow deer. The degree of mineralization of these deposits, which exhibited a high zinc content, was similar to that of the surrounding cartilage matrix. Intralacunar mineralization was also observed in some osteocyte lacunae. The intralacunar mineralization observed in antler cartilage and bone resembles the micropetrosis described for other bone types.

Based on the results of the present study, antlers are considered a promising model system not only for studying rapid bone growth and suppression of tumorigenesis during regeneration, but also for elucidating the processes involved in the mineralization of chondrocyte and osteocyte lacunae.

The following are the supplementary data related to this article.

**File S1:**
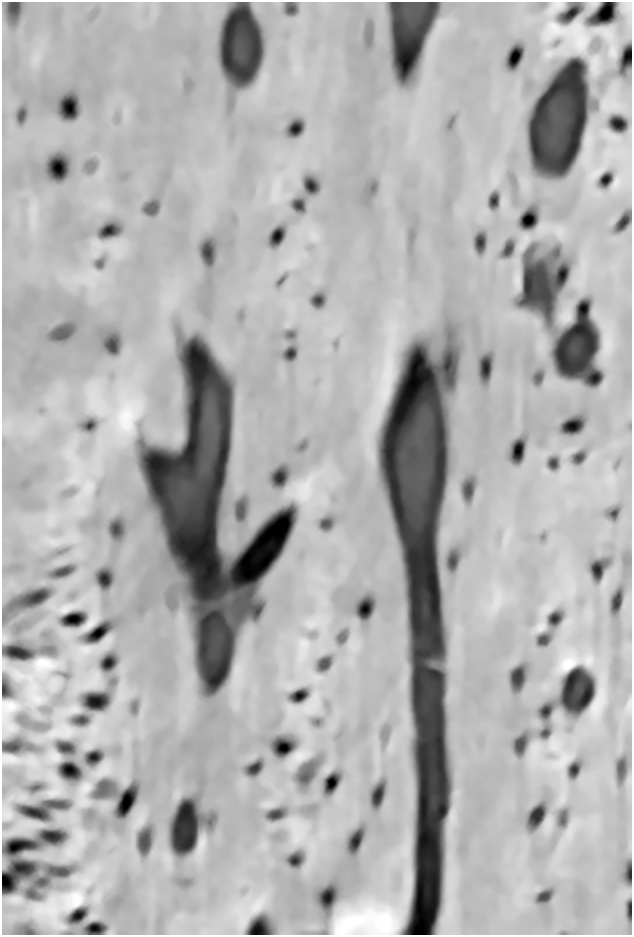
Stack of 151 slices from the SR-μCT scan of a bar obtained from the distal portion of a hard roe deer antler, 270 × 400 voxels per slice, field width of individual slice = 0.35 mm.

**File S2:**
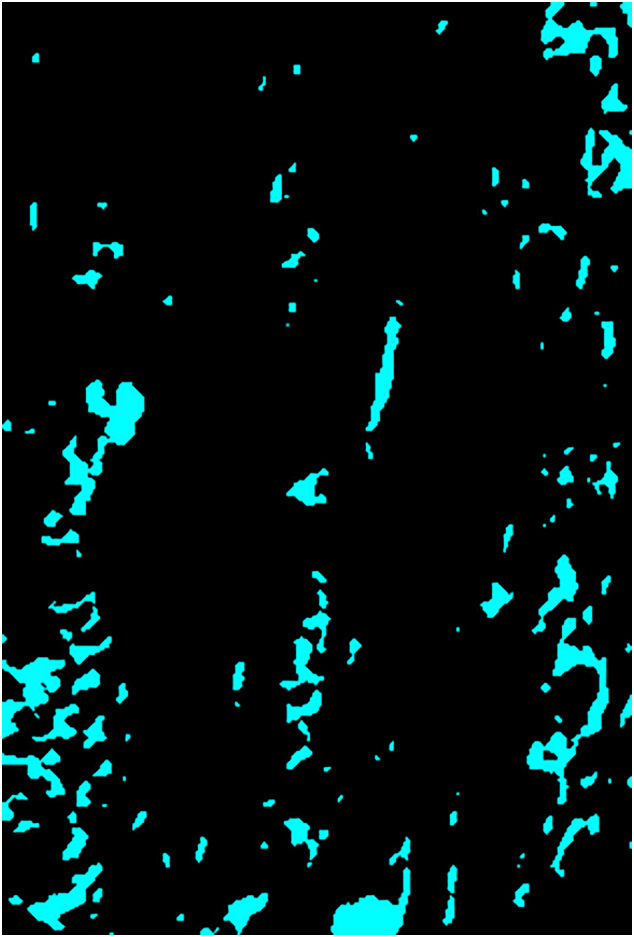
Segmented stack of 151 slices from the SR-μCT scan (cf. Fig. S1, cyan = calcified cartilage, black = bone, magenta = soft tissue spaces (voids).

**File S3:**
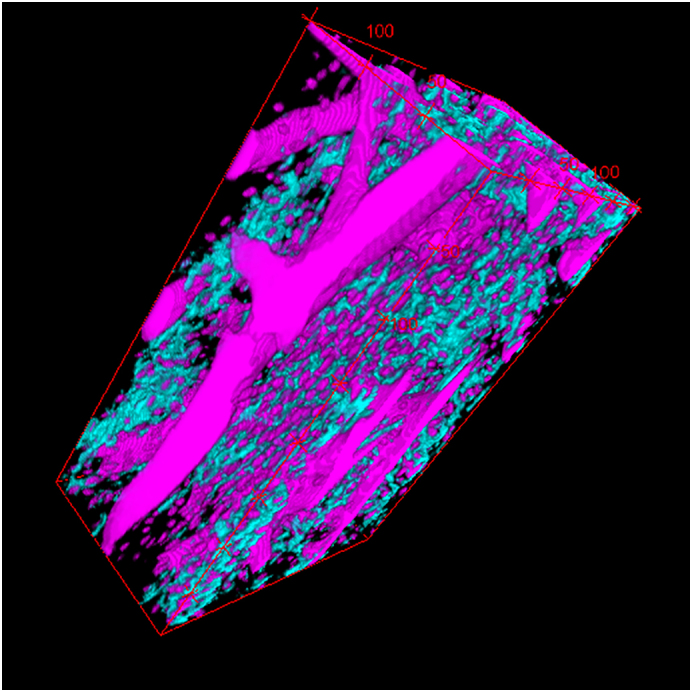
3D reconstruction of the segmented stack shown in Fig. S2, cyan = calcified cartilage, magenta = soft tissue spaces (voids).

## Availability of data and materials

The data supporting the findings of this study are available from the corresponding author upon reasonable request.

## CRediT authorship contribution statement

**Uwe Kierdorf:** Conceptualization, Methodology, Investigation, Formal analysis, Writing – original draft, Writing – review & editing. **Stuart R. Stock:** Methodology, Investigation, Formal analysis, Writing – review & editing. **Santiago Gomez:** Methodology, Investigation, Formal analysis, Writing – review & editing. **Olga Antipova:** Methodology, Investigation. **Horst Kierdorf:** Conceptualization, Methodology, Investigation, Formal analysis, Writing – review & editing.

## Declaration of competing interest

The authors declare that they have no conflicts of interest.
